# The ubiquitous interleukin-6: a time for reappraisal

**DOI:** 10.1186/1475-2840-9-62

**Published:** 2010-10-11

**Authors:** Enrique Z Fisman, Alexander Tenenbaum

**Affiliations:** 1 Sackler Faculty of Medicine, Tel-Aviv University, 69978 Tel-Aviv, Israel; 2 Cardiovascular Diabetology Research Foundation, 58484 Holon, Israel; 3 Cardiac Rehabilitation Institute, Sheba Medical Center, 52621 Tel-Hashomer, Israel

## Abstract

Interleukin-6 (IL-6) is a multifunctional cytokine regulating humoral and cellular responses and playing a central role in inflammation and tissue injury. Its effects are mediated through interaction with its receptor complex, IL-6Rβ (also known as gp130). It plays an important role in the pathogenesis of coronary artery disease and large quantities of IL-6 are found in human atherosclerotic plaques. IL-6 levels positively correlate with higher all-cause mortality, unstable angina, left ventricular dysfunction, propensity to diabetes and its complications, hypertension, obesity and several types of cancer. IL-6 levels augmentation demonstrates a remarkable parallel with another biomarkers reflecting harmful processes, like tumor necrosis factor alpha, interleukins 8 and 18, YKL-40, C reactive protein and resistin. Due to these facts, IL-6 was classified as a noxious interleukin. Nonetheless, there are several facts that challenge this usually accepted point of view. Since IL-6 has also anti-inflammatory activity, it seems reasonable to assume that favorable aspects exist. These aspects are two: 1. protection against bacterial infections, inactivating proinflammatory mediators, mitigating the course of septic shock and inducing the production of cortisol; and 2. influence on insulin sensitivity during exercise; this aspect is even more important. During exercise IL-6 is synthesized and released by muscles, with enhanced insulin action immediately at early recovery. Skeletal muscle may be considered as an endocrine organ; contracting muscles produce IL-6 and release it into the blood exerting its effects on other organs. The increase in circulating levels of IL-6 after exercise is consistent and proportional to exercise duration, intensity, muscle mass involved and endurance capacity. Thus, the fascinating possibility that the plenteous beneficial health effects of exercise could be ultimately mediated by IL-6 merits further elucidation. Interleukins were termed "good" or "bad", probably due to a tendency to see things in black and white, with no gray area in between. Calling IL-6 "a molecule with both beneficial and destructive potentials" would be a more equitable approach. In the literary creatures of Dr. Jekyll and Mr. Hyde, a good and an evil personality are found in the same individual. IL-6 playing the role of Dr. Jekyll is emerging; the time for IL-6 reappraisal is coming.

## 

Evidence continues to pile during the last two decades regarding the clinical relevance of laboratory predictors of pathophysiological events. These predictors are molecules, usually in the picogram (pg) range, called biomarkers. New and more specific biomarkers are currently isolated employing sophisticated bioinformatics approaches [1,2]. A biomarker is defined as a biochemical characteristic that may be objectively quantified and evaluated as an indicator of normal biological processes, pathogenic events, or responses to pharmacological or other therapeutic interventions. Thus, biomarkers are classified into 3 different types: Type 0, which estimates the emergence or advance of a disease, Type 1, which measures responses to therapeutic interventions, and Type 2, which may be employed as surrogate clinical endpoints [3,4].

In this context, interleukin-6 (IL-6) has gained a leading role. Its popularity is rooted on the fact that it can be defined either as a Type 0, 1 or 2 biomarker, depending on the given clinical setting. Thus, overlapping the 3 types of biomarkers, IL-6 attained a wide use in experimental and clinical studies. IL-6 is a multifunctional cytokine. It regulates humoral and cellular responses and plays a central role in inflammation and tissue injury. Its effects are mediated through interaction with its receptor complex, IL-6Rβ (also known as gp130) as a signaling subunit. This cytokine plays a very important role in the pathogenesis of coronary artery disease (CAD) [5]. Large quantities of IL-6 are found in human atherosclerotic plaques [6]. IL-6 levels are associated to higher all-cause mortality in elderly persons [7] and are elevated in patients with unstable angina compared with those with stable disease [8]. Moreover, patients with persistently elevated IL-6 levels demonstrate a worse in-hospital outcome following admission with unstable angina [9,10], as well as left ventricular diastolic dysfunction in both clinical trials [11] and experimental animal models [12]. We have also shown that in CAD patients with angina pectoris and/or healed myocardial infarction (MI), a significantly higher risk for future cardiac morbility and mortality was found in the upper IL-6 quintile (odds ratio, 3.44; 95% CI 1.57-8.13) after a mean follow-up period of 6.3 years [13]. IL-6 is also elevated in experimental MI models [14].

Additionally, IL-6 seems to embody a negative role in both main types of diabetes. After adjustment for age, gender, body mass index, waist-to-hip ratio, sports, smoking, alcohol consumption and other variables, IL-6 emerges as an independent early predictor of type 2 diabetes mellitus (T2DM), preceding its clinical onset [15]. Type 1 diabetes mellitus (T1DM) young patients - even in good glycemic control - show higher levels of IL-6 and fibrinogen than controls [16]. It has been shown that postmenopausal women with T1DM present higher serum bioactive IL-6 levels than matched healthy controls [17] and that subjects with diabetic foot show higher IL-6 values in comparison with T2DM patients without diabetic foot complication [18]. Additionally, IL-6 polymorphism seems to be a genetic susceptibility factor for the progression of diabetic nephropathy [19,20].

Besides its roles in the cardiovascular system and in regulation of glucose metabolism, IL-6 ubiquity is astonishing. Secreted by several types of cells, mainly macrophagues and T cells, the pleiotropic IL-6 is related to both immunoregulation and nonimmune physiological traits in most cell types and tissues outside the immune system [21]. Expressing a state of low grade chronic inflammation, IL-6 augmented levels have been reported to be directly related to the severity of sepsis [22], hypertension [23], poor survival among head and neck cancer patients [24], development of hairy cell leukemia [25], increased breast cancer risk in premenopausal women [26], intensity of inflammatory responses [27-29], obesity [30,31], increased insulin resistance [23,32-34], etc. In addition, IL-6 levels augmentation demonstrates a remarkable parallel with another biomarkers reflecting harmful processes, like tumor necrosis factor alpha (TNFα) [11,35], interleukins 8 and 18 [5,36], YKL-40 [37], C reactive protein (CRP) [38-40], resistin [41], etc. A comprehensive review of IL-6 numerous and complex biological effects is beyond the scope of this article.

Based on the above facts, IL-6 appears to be involved in a long series of deleterious actions, and it was classified as a noxious or "bad" interleukin [5,42]. Nonetheless, there are several facts that challenge this usually accepted point of view. A reconsideration is needed, as detailed hereinafter.

## Curtails of interleukin research

The concept of IL-6 being a damaging factor has growth mainly on the basis of statistical correlations in clinical studies, in vitro cell culture studies at supraphysiological levels of IL-6 and animal models employing mice [43].

Several important points should be taken into consideration when performing interleukin research: 1) increased levels of a given IL, presenting statistical correlation with disease, does not necessarily imply causation; 2) these compounds are characterized by substantial redundancy in that different interleukins have similar and overlapping functions; 3) interleukins may stimulate secretion of other interleukins, enhancing or inhibiting each other; 4) interleukins possess 'paradoxical' effects, expressed as protective properties regarding a given system, whereas they may damage another system, and 5) protective or noxious effects of a given interleukin may be concentration-dependent [5,42].

These general rules also pertain specifically to IL-6. Thus, studies "blaming" IL-6 based on statistical analyses or cell culture at high IL-6 concentrations could be partially biased by the mentioned intrinsic drawbacks. Regarding mice studies, it should be pointed out that comparative genomics research demonstrated that mouse and human IL-6 share only 42% aminoacid sequence identity [21]; hence, in this particular case extrapolation of mice findings to humans requires extreme caution.

Another problem that jeopardizes IL-6 research and may intricate IL-6 use as a bedside laboratory tool is the variability of its values. The established normal values are 1 pg/ml [44,45], but they increase at several settings like a single high fat meal [46] physical activity [47], normal menstrual cycle [48], acute hyperglycemia [49] or during and after surgery [50]. Pregnancy represents a good example of this inconsistency: median values of 129 pg/ml were registered at delivery, decreasing to 58 pg/ml immediately afterward [51]. Moreover, during sepsis IL-6 may reach even much more higher values [52].

## Beneficial effects

Besides its proinflammatory properties, IL-6 and IL-6-regulated acute-phase proteins show also anti-inflammatory activity [53,54]. Then, it seems reasonable to assume that IL-6 is not such a bad guy. We will briefly comment two aspects related to IL-6 favorable actions: bacterial infections and its particular influence on insulin sensitivity during exercise.

The protective IL-6 effects in infections were described almost three decades ago. Sometimes the interpretation of the results may be controversial since it is debatable whether in sepsis IL-6 represents an inflammation marker and/or a mediator of immune defense responses. Despite discordant results, some issues seem to be rather consistent. In neonatal mouse models of Group B streptococcal disease IL-6 decreases TNFα production, as well as the expression of TNF receptors in macrophages [55,56]; exogenous administration of IL-6 improved survival and complete inhibition of IL-6 resulted in a more rapid mortality [56]. IL-6 inactivates proinflammatory mediators and mitigates the course of septic shock [57]; it also induces the production of adrenocorticotropin and, in turn, cortisol, which is a potent anti-inflammatory hormone [58]. In mice infected with the intracellular pathogen Listeria monocytogenes, recombinant mouse IL-6 injected intraperitoneally before infection protected mice in a dose-dependent manner, resulting in decreased bacterial numbers in the spleen and liver; IL-6 played a role in early priming of the immune response to infection [59]. Anyway, it must be pointed out that these encouraging results are partially overshadowed by the lack of controlled clinical studies.

The second aspect - insulin metabolism during exercise - is even more important. The overwhelming in vitro findings linking IL-6 to increased insulin resistance may lack clinical relevance as in vivo human studies demonstrate that neither splanchnic glucose output nor isotopic tracer determined endogenous glucose production are increased by acute infusion of recombinant human IL-6 (rhIL-6) [43,60]. During exercise IL-6 is synthesized and released by skeletal muscle [61] and its plasma concentrations may be increased as much as 100-fold [47] - in parallel with the intensity and duration of exercise - this represents a far greater increase than that of any other cytokine that has been measured [61]. In this context, it was shown that IL-6 is rapidly released into the circulation following exercise [47], with enhanced insulin action immediately at early recovery. The improved insulin sensitivity is probably mediated by adenosine monophosphate-activated protein kinase (AMPK) [62,63].

In addition, rhIL-6 infusion during a hyperinsulinemic-euglycemic clamp in healthy humans does not effect the insulin-mediated suppression of endogenous glucose production, while increasing glucose infusion rate [64]. Exercise-induced improvement of insulin sensitivity is mainly a local phenomenon, occurring primarily in the exercised, rather than the rested, muscles. This was confirmed from experiments using both rodent and human models where muscles in only one limb have performed work prior to evaluation of insulin action in both limbs [63,65]. In both types of trials the prior exercised leg takes up glucose to a far greater extent and with enhanced insulin sensitivity compared with the rested leg. The changes may be of small magnitude, but over time they become important at the whole body [63].

It has been demonstrated that signaling pathways from contracting muscles to other organs are not solely mediated by the nervous system, since electrical stimulation of paralyzed muscles in spinal-cord-injured patients induces many of the same physiological changes as in neurological healthy individuals [66]. Therefore, it was clear that some humoral factor must exist. IL-6 fulfils the criteria of being that factor, via AMPK activation [47,54,64].

## Concluding comment

One of the more important proofs concerning the crucial importance of IL-6 emerges from an experimental rodent genetic model dated 3 quinquenniums ago. A targeted disruption of the IL-6 receptor complex gp130 was performed. Embryos homozygous for the gp130 mutation showed hypoplastic ventricular myocardium, reduced numbers of pluripotential cells - mainly hematopoietic progenitors - anemia and several additional abnormalities. The results indicate that gp130 plays a crucial role in myocardial development and hematopoiesis during embryogenesis [67].

Undesirable effects of IL-6 paharmacological blocking maybe reflected also in a clinical routine setting. In recent years, tocilizumab, a humanized anti-IL-6-receptor monoclonal antibody, was developed and successfully used for the treatment of rheumatoid arthritis and juvenile idiopathic arthritis [68]. Common adverse events of the therapy were gastrointestinal, nasopharyngeal, and upper-respiratory-tract infections. More serious adverse events - an anaphylactoid reaction and a gastrointestinal hemorrhage - were also reported [69]. These events are probably related to IL-6-receptor inhibition.

The key IL-6 positive action is its relationship with physical activity via enhancement of insulin-stimulated glucose disposal in humans in vivo [64,70]. Skeletal muscle may then be considered as a newly discovered endocrine organ; contracting muscles produce IL-6 and release it into the blood [43,47,71] exerting its effects on other organs in a hormone-like fashion. The increase in circulating levels of IL-6 after exercise is a consistent finding, proportional to exercise duration, intensity of effort, the muscle mass involved in the mechanical work and the endurance capacity [71]. Thus, the fascinating possibility that the plenteous beneficial health effects of exercise could be ultimately mediated by IL-6 merits further elucidation.

Interleukins were termed or classified as "good" or "bad" [5,13,72], probably due to a tendency to see things in black and white, with no gray area in between. Calling IL-6 "a molecule with both beneficial and destructive potentials" [73] would be a more equitable approach. In the literary creatures of Dr. Jekyll and Mr. Hyde, a good and an evil personality are found in the same individual [74]. IL-6 playing the role of Dr. Jekyll is emerging; the time for IL-6 reappraisal is coming.

## Abbreviations

AMPK: monophosphate-activated protein kinase; CAD: coronary artery disease; CRP: C reactive protein; IL-6: interleukin-6; TNFα: tumor necrosis factor alpha; T1DM: type 1 diabetes mellitus; T2DM: type 2 diabetes mellitus.

## Competing interests

The authors declare that they have no competing interests.

## Authors' contributions

Both authors have equally contributed in the conception and drafting of the manuscript. All authors read and approved the final manuscript.
